# Cardiovascular Implantable Electronic Devices Enabled Remote Heart Failure Monitoring; What We Have Learned and Where to Go Next

**DOI:** 10.3390/jcdd10040152

**Published:** 2023-03-31

**Authors:** Solmaz Assa, Kevin Vernooy, Antonius M. W. van Stipdonk

**Affiliations:** 1 Department of Cardiology, Treant Zorggroep, 7824 AA Emmen, The Netherlands; s.assa@treant.nl; 2 Department of Cardiology, Cardiovascular Research Institute Maastricht (CARIM), Maastricht University Medical Centre, 6229 HX Maastricht, The Netherlands; kevin.vernooy@mumc.nl

**Keywords:** heart failure, cardiac implantable electronic devices, remote monitoring, thoracic impedance, HeartLogic, triage HF

## Abstract

Despite recent developments, heart failure (HF) remains to be a great burden to the individual patient, entailing major morbidity and mortality. Moreover, HF is a great burden to overall healthcare, mainly because of frequent hospitalizations. Timely diagnosis of HF deterioration and implementation of appropriate therapy may prevent hospitalization and eventually improve a patient’s prognosis; however, depending on the patient’s presentation, the signs and symptoms of HF often offer too little therapeutic window to prevent hospitalizations. Cardiovascular implantable electronic devices (CIEDs) can provide real-time physiologic parameters and remote monitoring of these parameters can potentially help to identify patients at high risk. However, routine implementation of remote monitoring of CIEDs has still not been widely used in daily patient care. This review gives a detailed description of available metrics for remote HF monitoring, the studies that provide evidence of their efficacy, ways to implement them in clinical HF practice, as well as lessons learned on where to go on from where we currently are.

## 1. Introduction

Cardiovascular implantable electronic devices (CIEDs) have been used in heart failure (HF) patients with great success in either the prevention of sudden cardiac death or the treatment of dyssynchrony in HF [1,2]. In order to manage CIEDs, regular check-ups of hardware and tailored adjustments of programming are needed. These check-ups traditionally focus on device functioning, the early recognition of battery depletion, and potential device-related complications caused by either failure of a device component (e.g., leads) or the need for adjustment of device settings to the patient-specific situation. The advancement of remote connectivity has made it possible for these check-ups to be scheduled remotely since the early 90s. Together with the societal development of connectivity, remote monitoring of CIEDs has rapidly found its place next to routine outpatient follow-up of these devices [3]. 

Simultaneously, CIEDs’ capabilities to recognize bodily or cardiac changes related to HF, such as intrathoracic impedance, heart rate variability, nighttime heart rate, patient daily activity, and atrial and ventricular arrhythmias, have expanded [4]. All of these metrics have been shown to be associated with real-time physiological and pathophysiological (HF) status [5] and may therefore provide valuable information that can be used in the management of HF. Presumably, the early recognition and management of worsening HF events may therefore prevent HF hospitalization and even HF-related mortality [6,7]. Research into the CIED-facilitated telemonitoring of HF has taken flight since the 90s. However, large, randomized trials have shown contradictory results and currently, there is little evidence that structured CIED-facilitated HF monitoring reduces admissions for HF or mortality. Therefore, current ESC guidelines suggest that monitoring by CIEDs may be useful for detecting arrhythmias such as AF but still do not recommend routine use of CIED-based telemonitoring to prevent worsening HF events [8]. 

The remaining burden of HF admissions on individual patient quality of life and prognosis, as well as the burden on overall healthcare resources, necessitates the further evaluation of the potential value of CIED-facilitated HF monitoring. With the recent emergence and success of dedicated implantable monitoring devices (pulmonary artery pressure monitoring systems) in reducing WHF events [9,10], ascribed to the very early recognition of pathophysiologic changes in HF, the value of CIED-based monitoring of similar pathophysiologic changes deserves continued attention. Especially, as the information coming from these devices does not necessitate the implantation of (another) device at increased risk of complications and additional healthcare costs. In the current review we provide a detailed overview of the background and evidence on CIED-derived metrics that can individually, or together facilitate the goal of cost-effective device-based HF telemonitoring. 

## 2. Single Sensor Remote Monitoring

### Intrathoracic Impedance

**Figure 1:**
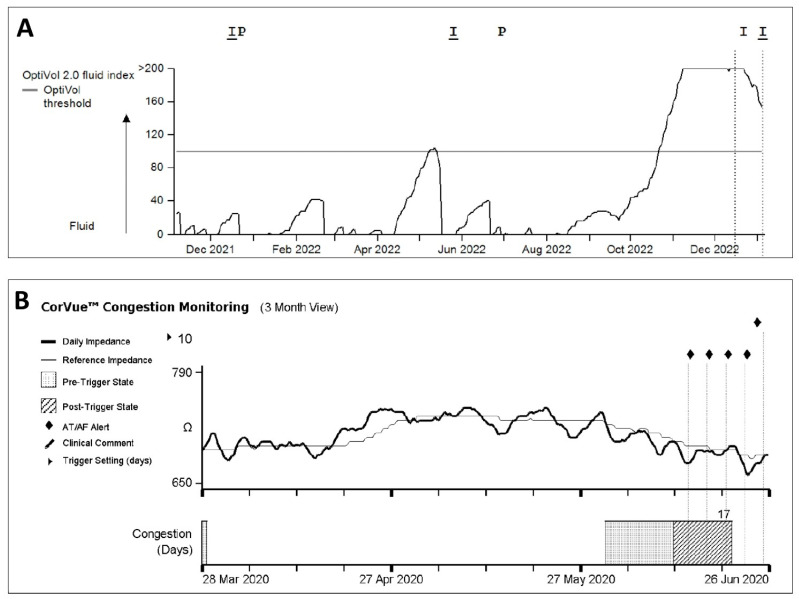
Example of (**A**) Optivol^TM^ and (**B**) CorVue^TM^ algorithm trends available in Medtronic and Abbott CIEDs, respectively.

Pulmonary congestion can increase intrapulmonary impedance. Due to the accumulation of intrathoracic fluid during pulmonary congestion, electrical currents generated by the CIED leads can be better conducted which is thus correlated with reduced intrathoracic impedance. A minute ventilation sensor uses a constant current with an asynchronous measurement frequency of 16 Hz; the resulting voltage is calculated as the intrathoracic impedance. Using averaged measurements at set hours each day, each value is then compared to a reference value (the difference is referred to as ‘fluid index’) and can thus be used to trigger an alert when exceeding a difference from this reference using a programmable threshold. As wound healing after CIED implantation may influence measurements, a 30-day period is taken into account before using measurements. In order to accommodate long-term impedance trends, the reference will be reobtained monthly. Measurement of thoracic impedance is integrated into ICD devices of all existing manufacturers, such as the Optivol^TM^ algorithm in Medtronic devices, the CorVue^TM^ algorithm in Abbott devices, and the BIO-Link^TM^ algorithm in Biotronik devices. In Boston scientific devices, the thoracic impedance measurement is part of a multi-sensor algorithm (HeartLogic), which will be discussed later. An example of the presentation of the intrathoracic impedance trends in the Optivol^TM^ and CorVue^TM^ algorithms is presented in Figure 1. 

In the observational MID-HeFT study [5], Yu et al. suggested an algorithm using intrathoracic impedance for the detection of the warning window before HF hospitalization (Yu et al., 2005). In their study, 34 patients with NYHA II-IV HF were prospectively included and followed during the chronic phase (outpatient) and acute phase (hospitalization) of HF. Intrathoracic impedance was measured from RV coil to device can and averaged for eight times 2 min measurements from noon to 5:59 PM (having the best correlation with HF hospitalization data). During hospitalization, measurements were more frequent. A threshold of 60 Ω (found in the algorithm development dataset) was found to have a 77% sensitivity, with 1.5 false positives and a warning of 13.4 days before HF hospitalization, in the validation dataset. During HF hospitalization, thoracic impedance increased because of diuretic therapy (62 ± 9.8 Ω to 72 ± 7.9 Ω). There was a strong correlation between intrathoracic impedance, pulmonary wedge pressure (r = −0.61; *p* < 0.001), and total negative fluid balance during hospitalization (*p* < 0.001). These findings were the initial steps in the development of the Optivol^TM^ algorithm in Medtronic devices. 

Subsequently, the Fluid Accumulation Status Trial (FAST) [10], a multicenter, prospective, and double-blinded study with the inclusion of 156 chronic HF patients and NYHA II-IV, confirmed the Optivol algorithms’ performance at a sensitivity of 74% and false positive rate of 2.08 per patient-year for detection of worsening HF events, even though worsening HF events in this study were defined broader, as HF hospitalizations, HF emergency department (ED) visits, or HF-related unscheduled office visits (Table S1). Two other prospective, observational studies, the InSync Sentry study [11], and the Insync Sentry Optivol^TM^ for the prediction of HF (SENSE-HF) 12 study were conducted to evaluate the diagnostic properties of the Optivol^TM^ algorithm in larger cohorts. The InSync Sentry study11 included 373 patients with HF implanted with a CRTD/ICD device. The majority of patients (90%) were in functional class II-III. The study used the Optivol algorithm to trigger an audible alarm from the CIED to alert the patient when the fluid index passed the nominal 60 Ω. A subsequent evaluation at the outpatient clinic would then follow for evaluation of HF deterioration, defined as mild to severe HF symptoms with or without an ED visit and HF hospitalization. The median time between alert onset and clinical evaluation was 3 (2–6, max 14) days. After correction for multiple events per patient, adjusted alert sensitivity for HF deterioration was 60% (95% CI 46–73%) (Table S1). The average monthly rate of false-positive alert events was 0.2 per patient-year. The SENSE-HF study [12] was a prospective, multicenter, observational study and included 501 chronic HFrHF patients with previous HF hospitalization in the last 12 months and the majority had NYHA II-III HF symptoms at inclusion. This study was performed in three phases: phase I started 34 days post-implant and lasted for the first 6 months. Both the patient and physician were blinded to the Optivol data. The aim was to assess the sensitivity and PPV of threshold crossing in the prediction of HF hospitalization with signs and symptoms of pulmonary congestion. In phase II, the physician had access to Optivol data and an audible alert was programmed upon which patients had to consult the physician. In both phases II and III, the PPV of the first alert in the prediction of worsening HF with signs and symptoms of pulmonary congestion was evaluated. During phase I, the sensitivity of crossing the threshold was 20.7% for the detection of HF hospitalization, and the sensitivity for the prediction of HF deterioration during phases II and III was 39.0% (Table S1). These values are lower than the sensitivity rates reported earlier. A post hoc analysis showed that the sensitivity rate was dynamic during phase I as it was 5.3% in patients experiencing HF hospitalization during 34–63 days after implantation and increased to a maximum of 42.1% in patients with HF hospitalization after 148 days post-implantation. The event rates were also higher early after implantation and that indicates a non-euvolemic status in a group of patients during this period with, as a result, a lower initial average daily impedance which may have affected the reference impedance and calculated fluid index and thus sensitivity rate. Moreover, different definitions of HF events and analysis of data may also have an impact on overall sensitivity during phase I, II, and III. 

Meanwhile, lower sensitivity in InSync Sentry [11] study may also be explained by the application of a significantly different endpoint than the original MID-HeFT study [5], with the possible underestimation of the eventual event rate, as physician assessment of HF deterioration occurred quite early after an alarm (median of 3 days after an alert), whereas the MID-HeFT study showed a median of 13 days between Optivol algorithm activation and the appearance of first HF symptoms.

Nevertheless, these studies showed that the implementation of the Optivol algorithm may (at least in a subgroup of patients) facilitate the detection of impending HF deterioration. An Optivol-alert triggered intervention may therefore reduce HF hospitalization or associated morbidity and even mortality. This was tested in two subsequent randomized controlled trials. 

In the prospective, randomized controlled DOT-HF study [13], patients with chronic HF (NYHA II-IV) were included within 6 months after implantation of an ICD/CRTD device. All patients had an HF hospitalization within 12 months of CIED implantation. The intervention included an Optivol alert (audible- or non-audible patient alert) to trigger a patient–physician (in-office) contact with a thorough review of the OptiVol and Cardiac Compass (other associated HF CIED parameters; see later) and clinical evaluation with a subsequent standardized intervention algorithm. The primary endpoint was all-cause mortality or HF hospitalization. The trial was terminated because of slow enrollment. A total of 335 patients were randomized to the Optivol-alert arm (168 patients) and to a control arm (167 patients). During the follow-up period of 14.9 ± 5.4 months, there was a numerical but not significant increase in primary endpoint events (combined all-cause mortality and HF hospitalization) in the Optivol-alert arm as compared to the control arm (HR 1.52, CI 0.97–2.37, *p* 0.063) (Table S2). There was no difference in the death rate between groups (19 in the Optivol-alert arm vs. 15 in the control arm; HR 1.24, CI 0.63–2.44, *p* 0.54). However, the number of unscheduled hospitalizations was significantly higher in the Optivol-alert arm vs. control arm (60 vs. 36, HR 1.79, CI, 1.08–2.95: *p* 0.022). Interestingly, there was no difference between the two groups in the incidence of hospitalizations not preceded by an Optivol alert. The number of unscheduled outpatient visits was also higher in the Optivol-alert arm (250 visits) vs. the control arm (84 visits) (*p* < 0.0001) 

In 2016, Bohm et al. published the OptiLink HF (Optimization of HF Management using OptiVol^TM^ Fluid Status Monitoring and CareLinkTM) study [14], a prospective, multicenter, randomized, and unblinded study which included chronic HF patients in NYHA II-III with an ICD/CRTD device, and either prior HF hospitalization, recent diuretic treatment, or recent BNP increase. Patients were randomized to automatic transfer of fluid index alerts or standard care. A protocol-specified intervention followed every transmitted alert which included a review of the HF-specific device data and contact with the patient within 2 days to evaluate the clinical condition and HF status. After 18 months, there was no difference in the primary outcome (composite of all-cause mortality and CV hospitalization) between the two groups (event-free survival 59.7% in the intervention group vs. 56.1% in the control group, HR, 0.87, CI 0.72–1.04, *p* = 0.13). The secondary outcome combined all-cause mortality and HF hospitalization was significantly lower in the intervention group (*p* = 0.03). In this study only 30.1% of fluid index alerts were followed by a medical action and 26% led to medication change. 

An important point worth considering is that in all the above studies intrathoracic impedance was measured by a single vector from the RV coil to the can, as was suggested by MID-HeFT trial5. Using different impedance vectors from RV and LV leads may evaluate the larger region of the lung and increase sensitivity for the detection of HF events. Therefore, the CorVue algorithm was developed in St Judes (currently Abbott) devices which applies combined vectors to estimate intrathoracic impedance. Initial results in the retrospective feasibility study showed that when combined RV ring → Can + RV coil → Can and LV ring → Can + RV coil → Can were used in ICD and CRTD devices, respectively, this algorithm was able to predict pulmonary congestion with a sensitivity of 71.4%, which was superior to conventional single vector (RV coil → can) (sensitivity 57.1%) [15]. 

This multivector algorithm was subsequently evaluated in the DEFEAT-PE trial [16], a prospective multicenter study. A total of 144 HF patients with previous HF hospitalization and ICD/CRTD devices were included and the majority of them were in NYHA class II-III. Intrathoracic impedance was measured every 2 h and the average was used to calculate the daily impedance. An average of daily impedance over time was used to determine the reference impedance. A registered daily impedance below the reference impendence was marked as a “sensed state of congestion” which when present for several days could mark an “algorithm-detected event”. If the first day of the algorithm-detected event was <30 days before clinical pulmonary congestion, then the alert was considered positive. 

The ICD algorithm with RV ring → Can + RV coil → Can and nominal threshold of 14 days (FDA approved) showed a sensitivity of 26.2% for the prediction of a clinical event with pulmonary congestion. Likewise, the CRTD algorithm with LV ring → Can + RV coil → Can and a nominal threshold of 14 days showed a sensitivity of 17.6%. The performance of the CorVue algorithm in the entire cohort showed a sensitivity of 21.6% (Table S1). In conclusion, when prospectively studied in a larger group of patients, the CorVue algorithm did not show improved sensitivity for the prediction of worsening HF (defined as clinical events with pulmonary congestion) as compared to a single vector algorithm. 

Altogether, thoracic impedance measurements are a standalone measurement to detect early conditions of worsening HF and hence an opportunity to prevent HF events that lack robust evidence of diagnostic properties that can help guide early management of worsening HF. Apart from modest sensitivity to detect worsening HF and future HF events, its use as a trigger for management alterations has not proven any benefit to the patient, but an increase in healthcare burden. 

These findings should be seen in the light of several limitations of both the thoracic impedance measurements itself, as well as the design of the studies that have tried to prove its value. Possibly the diagnostic properties of this algorithm are affected by factors such as HF worsening presenting without evident pulmonary congestion and the change of intrathoracic impedance due to other reasons besides congestion (such as acute arrhythmia, acute anemia, pneumonia, pericardial or pleural perfusion, and COPD4). Moreover, all the above-mentioned studies were carried out by application of a nominal 60 Ω threshold. Defining a higher threshold of 120 Ω in a small study [17] showed higher specificity but also lower sensitivity. Probably, the application of a personalized threshold for alerts will produce the best diagnostic accuracy. With respect to the action following the measurement of a relative decrease in thoracic impedance, the implementation of audible alerts for patients seems not to be a proper tool to trigger the adjustment of HF management as it may lead to increased healthcare burden by stressing both the patient and caregiver. The intervention studies were designed to use thoracic impedance measurement as a tool to reduce HF endpoints and use a physician consultation to trigger treatment adjustments. However, when the alerts trigger on average 13 days before any evidence of complaints or signs of WHF show, short-term evaluation by a physician may not be the right next step. The same issue arises when considering the definition of the endpoint in the trials mentioned. Robust endpoints such as hospitalization or the need for diuretic therapy, rather than the detection of HF signs and symptoms at physician evaluation should be taken as endpoints. After all, if diagnostic accuracy would be considered adequate, the alert should trigger immediate treatment adjustments rather than another evaluation (known to be flawed). However, as doubts about diagnostic accuracy remain, trials to trigger immediate treatment adjustment may entail considerable safety issues. Hence, the first step to take for further assessment of the usability of CIED-facilitated HF monitoring should be to improve its diagnostic accuracy. 

## 3. Multisensor Remote Monitoring

Thus far, single parameter monitoring by the application of intrathoracic impedance seemed to be of limited value in the prediction and prevention of clinical HF events with only modest sensitivity and worse specificity. The above-mentioned findings have triggered the field of research to study the use of a combination of intrathoracic impedance measurement with other physiologic parameters made available by CIED monitoring, to provide additive value to the diagnostic properties, especially lowering the number of false-positive alerts. Parameters such as atrial/ventricular arrhythmia, patient activity level, heart rate variability (HRV), heart rate during the night, and ICD therapy may increase the sensitivity and/or specificity of CIED-based HF monitoring.

Whellan et al. [18] included 694 CRT patients in the prospective, multicenter observational PARTNERS HF study to evaluate the prediction of HF deterioration using combined HF diagnostic information (Table S1). The Medtronic Cardiac compass HF device diagnostic parameters and algorithm were used. The algorithm was considered positive when the fluid index was >100 Ω or any two of the following criteria were present: long AF duration (>6 h in one day or >23 h during 7 consecutive days), rapid ventricular rate during AF (≥90 bpm), high fluid index (≥60), low patient activity (≤1 h during 1 week), high night heart rate (>85 bpm for 7 consecutive days), low HRV (<60 ms for at least 5 days), low CRT pacing (<90% for 5–7 days), or ICD shocks (≥1 shocks). The algorithm grants each individual component of the algorithm equal value in its contribution to trigger an alert. The algorithm was based on a development set of 819 patients from a separate clinical registry trial. HF diagnostic data were gathered from 60 days post-implantation onward. The primary endpoint was HF hospitalization with pulmonary congestion and the mean follow-up was 11.7 ± 2.0 months. Events were considered related to the positive algorithm when the event occurred in the preceding evaluation period. This was performed in semi-monthly, monthly, and quarterly assessment periods, and at each moment of evaluation, HF diagnostic criteria were assessed retrospectively and the occurrence of the first HF event was observed prospectively. Patients with a positive diagnostic algorithm in the periodic evaluations had a significantly increased risk of hospitalization with pulmonary congestion within the next month (HR 5.5, 95% CI 3.4–8.8). The risk of hospitalization with pulmonary congestion remained high in patients with positive diagnostic algorithms even after correction for clinical factors that possibly affected the primary outcome (HR 4.8, 95% CI 2.9–8.1). In comparison, the application of only a high fluid index above 60Ω had a less predictive effect (2.7-fold increased risk of HF hospitalization). Risk-quantification capability of the above algorithm was higher when the interval duration between assessments was shorter (semi-monthly HR of 6.9 vs. monthly HR of 5.5 vs. quarterly HR of 3.1). 

The SELENE-HF [19] study was another prospective, observational, multicenter, event-driven study with an enrolment of 918 heart failure patients (LVEF ≤ 35%, NYHA II-III) with a BIOTRONIK ICD/CRTD which used combined remote monitoring parameters and a baseline risk stratifier (Seattle HF model) to develop a predictive algorithm. The remote monitoring parameters included 24 h heart rate, night heart rate, heart rate variability, 24 h activity, atrial high-rate episode (AHRE) burden, amount of PVCs per day, and thoracic impedance. Patients were randomly divided into a derivation and a validation group. The primary endpoint was first adjudicated post-implant (≥30 days) hospitalization for worsening heart failure (non-elective admission with signs and symptoms of worsening heart failure and requirement of administration of intravenous diuretics). The secondary endpoint was a composite of any (first or subsequent) hospitalization, outpatient intravenous intervention, or death related to worsening heart failure. Patients were followed twice a year, with in-hospital evaluation. The algorithm was developed based on the occurrence of the primary endpoint in the derivation group. The predicting index was calculated daily in each patient and when exceeded a nominal threshold for several consecutive days an alert was triggered. After each alert, the nominal threshold was replaced by a lower recovery threshold and the difference was called the offset of the recovery threshold. A true-positive alert was defined whenever an index did not fall below the recovery threshold between the alert and the event, otherwise the alert was canceled. In the derivation group, the index showed a C-statistics of 0.89 (95% CI: 0.83–0.95) with an odds ratio of 2.73 (CI 1.98–3.78) for first HF hospitalization per unitary increase in index value (*p* < 0.001). In the validation cohort, the sensitivity of prediction of the primary endpoint with a nominal threshold of 4.5 was 65.5% (CI 45.7–82.1%) with a median alert time of 42 days and a false alert rate of 0.69 alerts per patient-year. A lower nominal threshold (e.g., 3.5) increased the sensitivity (e.g., 72.4%) at the cost of a higher false alert rate (e.g., 1.07). The sensitivity for the prediction of the secondary endpoint at the nominal threshold of 4.5 and 3.5 was 54.8 and 64.5%, respectively, the median alerting time was 43–60 days, and the false alert rate was 0.67–1.05. Without the baseline risk stratifier, the sensitivity remained the same (65.5%) but the false alert rate increased by 10% to 0.76 per patient-year.

Subsequently, intervention studies tested the combined algorithms’ value in HF management. In the EVOLVO [20] study, a randomized, multicenter, open-label study, remote monitoring of 200 HF patients with ICD/CRTD was applied using Medtronic CareLink Network. Patients were randomized to “remote transmission on” (remote arm) for transmission of clinical management data (intrathoracic impedance, atrial arrhythmia, and delivered ICD shocks) via CareLink (and “audible notifications off”) or “remote transmission off” (standard arm) without access to CareLink but alerts were on for audible notification only. The CareLink website was checked at least once daily for new transmissions. During 16 months of follow-up, a comparable number of wireless notification and audible notifications was generated in the remote arm and standard arm (315, 2.5 events per year vs. 256, 2.40 events per year, respectively; CI, 0.89–1.23; *p* 0.602). However, the number of emergency department/in-office visits during follow-up was significantly lower in the remote arm as compared to the standard arm (75 vs. 117, respectively; IRR 0.65; CI 0.49–0.88; *p* < 0.005). Remote monitoring also reduced visits for HF worsening (48 vs. 92; IRR, 0.52; CI 0.37–0.75; *p* < 0.001), but visits for arrhythmia or ICD-related episodes were similar (*p* = 0.649). Overall healthcare use was significantly lower in the remote arm (4.40 events per year in the remote arm vs. 5.74 events per year in the standard arm; IRR 0.79; CI 0.71–0.89; *p* < 0.001). In the remote arm, 86% of the visits were considered necessary (resulting from an appropriate and clinically meaningful ICD alert) whereas in the standard arm only 53% of the visits were determined to be clinically meaningful (*p* < 0.001). The time-to-device data review was longer in the standard arm than in the remote arm (24.8 days vs. 1.4 days). Even though patients in the standard arm were instructed to contact the healthcare center in the case of symptoms or alerts, the authors observed that not all the alerts were followed by a clinic visit. In a prospective single-center randomized pilot study, Luthje et al. [21] evaluated the influence of remote monitoring in combination with wireless fluid monitoring using Optivol alerts. A total of 176 patients were randomized to either the remote arm (remote monitoring via CareLink ON including Optivol ON) or the control arm. Interestingly, HF or a history of HF hospitalization was not a prerequisite for inclusion. In case of a transmitted alert, patients were treated according to a prespecified clinical management protocol. The protocol dictated that a patient–physician in-office contact should follow, including a review of Cardiac Compass and clinical status. When defined as a true positive alert, hospital admission followed in the case of evident HF clinical symptoms, or outpatient treatment with diuretics was applied, when not so evident. Patients in the control arm were evaluated every 3 months via standard in-office visits. In contrast with the EVOLVO study, within 15 months of follow-up, the number of urgent care visits was higher in the remote group as compared to the control group (0.3 ± 0.50 vs. 0.10 ± 0.30 visits, respectively; *p* = 0.0332). However, there was no difference in time to HF hospitalization between the groups (HR 1.231; CI 0.621–2.438; *p* = 0.551) nor in the number of emergency department visits (RM group 0.10 ± 0.25 vs. control group 0.10 ± 0.23; *p* = 0.7295), time to first ICD shock (*p* = 0.512) and mortality (*p* = 0.502). One explanation could be due to the early detection of preliminary signs and symptoms of HF deterioration in the remote arm (and as a result early medical intervention) and this situation could sometimes improve spontaneously without any intervention, as may have happened in a group of patients in the control group. Therefore, it seems that the different management strategies of HF deterioration were the main reason for the different results in this study, whereas these did not have a meaningful clinical impact.

The first large, and only positive randomized controlled trial in CIED-enabled HF monitoring was performed by Hindricks et al. [22] in 2014. The IN-TIME21 study used a similar multiparameter telemonitoring strategy in a randomized, controlled, multicenter trial including 664 NYHA II-III HF patients with a Biotronik ICD or CRTD device implanted. Patients were randomly assigned to an automatic, daily remote-monitoring-based follow-up (333) versus standard care (331). Patients were initially blinded to treatment allocation (until contacted because of remote monitoring findings). A composite endpoint of worsening HF events (all-cause death, HF hospitalization, change in NYHA class, and change in patient global self-assessment) was used to evaluate the effect of remote HF monitoring. The management of the transmitted data was up to the clinical routine at each of the 36 study sites; however, a central monitoring unit was installed to make sure that awareness of predefined medical events was present at the study sites through email notifications. When physicians chose to contact patients triggered by remote transmission, a standardized telephone interview established the patient’s overall condition. There was no formal management protocol available to the physicians. During 1 year of follow-up, 238 patients (71%) in the telemonitoring group were contacted at least once. The central monitoring unit forwarded an overall 4.0 transmissions per patient-year. Transmissions triggered patient contacts in 71% of patients, corresponding to 2.1 contacts per patient-year, and these lead to 99 additional follow-up visits (19%, or 0.32 extra visits per patient-year). The primary composite worsening HF endpoint was met in 18.9% in the remote monitoring group and 27.2% in the standard care group (OR 0.63; 95% CI 0.43–0.90). Strikingly there was no significant reduction in HF hospitalization (*p* = 0.38), worsening NYHA class (*p* = 0.43), or worsened self-assessment score (*p* = 0.63), but there was significantly lower all-cause mortality in the telemonitoring group (3.4% vs. 8.7%; HR 0.36; 95% CI 0.17–0.74).

The subsequent, even larger, landmark trial in CIED enabled HF monitoring, the REM-HF study [23] was a randomized, multicenter, open-label study. A total of 1.650 HF patients (NYHA II-IV, majority NYHA II) who had a CIED (CRTP/D or ICD, implanted at least 6 months earlier) and optimal HF therapy were randomized to multiparameter CIED remote monitoring (weekly download from patients’ devices) or usual care. In this study, no specific alerts were triggered by the multiparameter CIED remote monitoring program, but rather changes in multiparameter trends were reviewed. This study’s design was different from previous studies, in that it included CIEDs from different manufacturers, with as a result, a small difference in the parameters available for remote HF monitoring. A procedural handbook was applied to guide physicians’ actions to the observed trends after the interpretation of the downloaded data, and the center’s remote monitor was enabled to coordinate management. The management change plan was consistent across the study and included drug change, primary care test (e.g., electrolyte estimation), specialist test, programming change, or specialist intervention. Median follow-up was 2.8 years (0–4.3 years) and there was no difference in the rate of the primary endpoint (combined death or first hospitalization for CV reasons) between the two groups (42.4% remote group vs. 40.8% usual care group, HR 1.01, CI 0.87–1.18, *p* = 0.87). The two groups were also similar in the occurrence of secondary endpoints such as all-cause and cardiovascular mortality (as mentioned in Table S2). This striking difference in results despite the even larger trial, can be explained by several differences. First, the patients were less sick at baseline (majority NYHA II in REM-HF), even though mortality was similar. Secondly, endpoints significantly differed. Using the hard endpoint of death or CV hospitalizations in the REM-HF study, compared to the use of NYHA of patient global self-assessment score in the IN-TIME study [21]. Where the main difference in the IN-TIME study was driven by all-cause mortality, HF hospitalizations were not affected in either study [21,22]. The largest difference in the two studies, however, was the striking difference in monitoring strategies: ‘alert based’ by a central-monitoring institute applied protocol versus physicians’ discretion multiparameter trend triggered management. It seems that the interpretation of multi-parameter trends by physicians simply fails to see trends that can be captured by protocolled, dedicated ‘alert-based’ monitoring. 

To overcome the limitations of human-based interpretation of this physiologic point-data or trends, Cowie et al. [7] used a Bayesian Belief Network (BBN) framework in a development set (data from OFFISSER [24], Italian Clinical Service [25] Project and CONNECT studies [26], 921 patients) and validation set (PARTNERS-HF18, FAST10, PRECEDE-HF, and SENSE-HF studies [12], 1310 patient), to develop and validate a multiparameter CIED based HF monitoring risk score for the identification of the patients at risk for HF hospitalization. The parameters included were intrathoracic impedance, AF burden, ventricular rate during atrial fibrillation (VRAF), ventricular tachycardia (VT) episodes, patient activity (ACT), day and night heart rate (NHR), and heart rate variability (HRV). In their study, each of the diagnostic elements included in the final risk score showed an individual capability of identifying patients at risk for HF hospitalization. The combined risk score referred to as HF Risk Score (HFRS) was calculated using the maximum measures from the last 30 days before HF hospitalization. A risk score < 0.054 was categorized as low HFRS, 0.054–0.20 as medium HFRS, and ≥0.20 as high HFRS. In the validation set, patients with a high HFRS were 10 times more likely to be hospitalized for HF in the next 30 days, when compared with a low-risk state.

HFRS was thereafter validated retrospectively in the RAFT trial [27], a multicenter, randomized, controlled trial in 1.798 patients with NYHA II-III heart failure which were randomized to ICD vs. CRTD to evaluate the effect on the primary endpoint of all-cause mortality or hospitalization for HF. Hospitalization for HF alone was a pre-specified secondary outcome. HFRS parameters were analyzed monthly and HF exacerbation was evaluated in the 30 days after monthly analysis. In 1.224 patients with devices capable of monitoring all the parameters in HFRS, the incidence of HF hospitalization was significantly higher when preceded by a high-risk HFRS the month before (2.61% per month, RR compared with low-risk months 10.7 (6.9–16.6)) vs. medium-risk (0.66%, RR compared with low-risk months 2.9 (2.0–4.4)) and low-risk (0.21%) months. 

In a recent prospective observational study (439 patients, 73% with heart failure) [28], it was shown that HRFS data are associated with all-cause mortality. In this study, the occurrence of all-cause mortality was higher in patients with at least one high HFRS episode than in patients who had never had a high HFRS (OR: 3.07, 95% CI: 1.57–6.58, *p* = 0.002). Moreover, there was more cardiovascular mortality in the high HFRS group than in the non-high HFRS group (10.3% vs. <4.0%; *p* = 0.03). Of interest is not only the HFRS state but also increased days spent in an HFRS state has been shown to be associated with increased mortality (OR: 1.00, 95% CI: 1.00–1.02).

**Figure 2:**
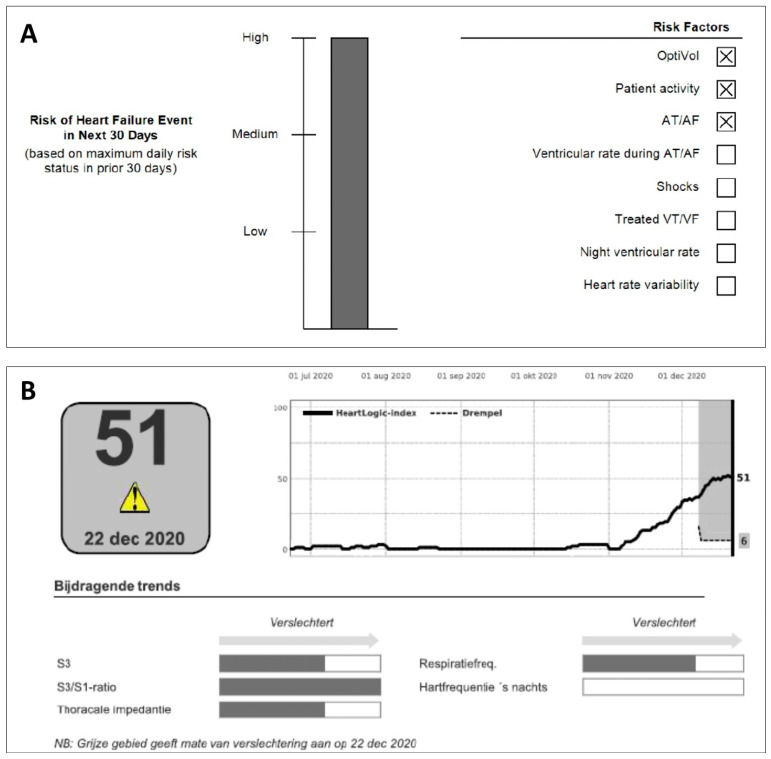
(**A**) TriageHF^TM^ and (**B**) HeartLogic^TM^ multisensory HF telemonitoring algorithm employed in Medtronic and Boston Scientific CIEDs, respectively.

The HFRS has been employed in Medtronic devices as the TriageHF^TM^ algorithm (Figure 2, Table 1).

In 2017 the prospective TRIAGE-HF trial was conducted [29] as a pilot study in 100 patients with heart failure (majority NYHA II-III, mean EF 31%) and a CRTD/ICD device. The primary objective of the study was to correlate high HFRS with signs and symptoms and behaviors associated with worsening HF. Patients were followed for 8 months and all patients with high HFRS and some of the patients with moderate HFRS (at the discretion of the caregiver) were actively contacted. During follow-up, 83% of patients with high HFRS had signs/symptoms of HF or non-compliance to medication, whereas this number was only 8% in patients with medium HFRS. Although not the primary goal of the TRIAG-HF study, patients contacted due to high HFRS triggered optimization of treatment in 54% of them. 

Consequently, the prospective cohort TRIAG-HF-Plus [30] aimed to evaluate the real-world accuracy of HFRS followed by telephone triage in high HFRS patients (TRIAG-HF plus pathway) in a large cohort of patients (231 patients with a Medtronic CIED, including CRTP and pacemakers). CareLink transmissions were reviewed every 3 months or earlier in the case of manual transmission by the patient or an activated Care Alert transmission. The data were then reviewed by a consultant cardiologist and patients at high HFRS were contacted by telephone for screening with standardized questions for worsening of heart failure (WHF). Those patients with characteristics of isolated WHF or in combination with an acute medical problem were identified as triage positive. In this single-center study, the sensitivity and specificity of a high HFRS were 98.6% (92.5–100.0%) and 63.4% (55.2–71.0%), respectively, with an overall accuracy of 74.7% (68.5–80.2%). Only one patient in the algorithm-allocated ‘negative’ group had symptoms and signs of WHF. A sample of low/medium HFRS patients were also contacted by telephone for screening and the results showed that 99% of patients had no features of WHF. 

An algorithm similar to Triage HF, named Heartlogic^TM^ has been developed by Boston Scientific. This algorithm includes different Boston-specific physiological sensors such as S1–S3 heart sounds (and the ratio of the two), thoracic impedance, respiration rate, ratio of respiration rate/tidal volume, nightly heart rate, and patient activity to predict worsening HF (Table 1). After a blanking period of 40 days to construct a patient-specific baseline, the data are transmitted to the remote monitoring system (LATITUDE, Boston Scientific). The HeartLogic index is updated daily and activates a remote alert when the index crosses the threshold of 16. Although not exactly known, the algorithm development strategy, briefly described in the MultiSENSE study describes individually HF event associated parameter-trends during the pre-event period modeled into a multiparameter set, such as the TriageHF^TM^ algorithm. 

The MultiSENSE [31] trial was an international, multicenter, non-randomized study in 974 HF patients (531 development group and 443 validation group) with CRTD devices. In the independent validation set, the HeartLogic algorithm had a sensitivity of 70% for the prediction of HF events (defined as hospitalizations or outpatient visits for IV therapy with HF as the primary diagnosis) with a UAR (unexpected alert rate) of 1.47 per patient-year and negative predictive value around 99.98%. An alert was activated at a median of 34 days before the impending HF event.

Several cohorts after the MULTISENSE study provided confirmatory results and suggested that the HeartLogic algorithm can be potentially useful for the prediction of HF deterioration and events.

In 2020, a prospective study of 104 HF patients (majority NYHA II-III) [32] was published which showed that the application of the HeartLogic algorithm produced 60% clinically meaningful alerts during follow-up, defined as alerts associated with HF events (including initial signs and symptoms of HF, HF hospitalization) or alerts that resulted in active clinical actions. The unexplained alert rate was 0.37 per patient-year and the false-negative rate was 0.05 per patient-year. 

Caló et al. [33] performed a multicenter study in 366 HF patients (majority NYHA II-III) with CIED and activated the HeartLogic algorithm. They showed that during a median follow-up of 11 months (25–75th percentile 6–16) IN alert state was associated with higher HF events (HF hospitalization) than the OUT alert state (HR 30.6, CI 13.0–71.9, *p* < 0.001). Similarly, more HF deaths were reported in the IN alert state than in the OUT alert state (HR, 11.45; CI 5.55–23.60; *p* < 0.001). Alerts accompanied by a clinical action were associated with lower HF event rates than those without any clinical actions (HR, 0.34; CI 0.12–0.96; *p* = 0.04). There was no difference between remote and in-office alert management. 

A different multicenter study by Treskes et al. [34] applied the HeartLogic algorithm to 68 HF patients (NYHA I-III). During 365 follow-up days after activation of the HeartLogic algorithm, the sensitivity and specificity for HF events (HF hospitalization or early signs and symptoms of HF resulting in application of IV diuretics or increased oral diuretics) were 90% (CI 77–97%) and 89% (CI 79–95%), respectively. The unexplained alert rate was 0.16. When comparing the period before (365 d) and after activation of the HeartLogic algorithm, there were fewer HF hospitalizations in the post-activation than pre-activation period (0.11 ± 0.04 vs. 0.39 ± 0.08 hospitalization per patient/year, respectively; *p* = 0.003). A cost-effective analysis in a subgroup of patients (Belgium center) showed a significant reduction in total cost per patient (€9958 difference; *p* = 0.025) and average hospitalization cost per patient (€9972 difference; *p* = 0.028) after activation of HeartLogic algorithm.

Recently, a prospective single-center study in 107 HF patients (NYHA I-III) [35] with CIEDs and activated HeartLogic revealed a sensitivity of 79% (CI 0.68–0.86) and specificity of 89% (CI 0.08–0.15) for the detection of early signs of fluid retention. The estimated positive and negative predictive values were 71% (CI 0.61–0.80) and 91% (CI 0.06–0.13), respectively. The false-negative rate was 0.17 events per year.

The higher sensitivity and specificity in the last two studies in comparison with the MULTISENSE study was most probably due to the different definition of HF events, as in the MULTISENSE [31] study only HF hospitalizations were counted as HF events, and the recent studies also included early signs and symptoms of HF deterioration. 

The HeartLogic algorithm is currently being investigated prospectively in two clinical trials: 

The PREEMPT-HF study (NCT03579641) is a prospective study that aims to evaluate the extended application of HeartLogic^TM^ sensor data for association with 30-day HF readmissions. The results of this study are expected in 2024. The MANAGE-HF trial (NCT03237858) is a multicenter, randomized, double-blind study which evaluates the clinical efficacy of the HeartLogic^TM^ HF diagnostic features in preventing HF deterioration. Phase 1 of this study has recently finished studying the clinical integration of HeartLogic^TM^ for managing patients with HF [36]. This open-label, single-arm study included 191 high-risk HF patients with a CIED with the HeartLogic algorithm activated, with HF hospitalization <12 months, unscheduled visit for HF exacerbation <3 months, or increased natriuretic peptides at baseline. After the transmission of an alert, clinicians were encouraged to follow an alert management guide that was developed by the MANAGE-HF steering committee. Alert management guide provided algorithms for clinical assessment of the patients with an alert and thereafter escalation of disease-modifying HF and/or diuretic therapy from the pre-alert treatment. At re-alerts, the treatment was maintained if the index was below the index in the prior week or escalated further if no improvement of the index was noted. Importantly the AMG explicitly stated that the absence of signs and symptoms of worsening HF does not justify an exception from escalation of therapy, after the exclusion of contra-indications. From a clinical perspective, 1.76 alert cases were noted per patient-year, with an average alert duration of 36 days. A total of 81% of alerts were followed by an attempt to contact patients (successful in 93% of attempts). At the initial alert, 64% of patients presented with at least one symptom of worsening HF. HF treatment was augmented during 74% of alert cases (89% diuretic changes and 33% disease modifying HF therapy) and patients followed by HeartLogic had a lower natriuretic peptide level at 12 months follow-up, compared to baseline 1316 [664–2856] to 743 [336–1681). The IN alert status was detected 30 days prior to 83% of HF-hospitalization and 77% of IV outpatient treatments. With respect to safety, there were 50 adverse events, of which 5 serious adverse events (7% and 0.7%) were possibly related to alert-triggered modifications (renal insufficiency, dizziness, and syncope) out of a total of 691 AEs. The conclusion of phase 1 of this study therefore was that implementation of HeartLogic in clinical care was safe and able to lower natriuretic peptide levels, as a surrogate marker of HF prognosis. Phase 2 of the study, the randomized, multicenter, double-blind part of the study, will evaluate the clinical effectiveness of HeartLogic^TM^ in alert-directed HF care on patient outcomes. This study is expected to be completed by 2025. 

Altogether, initial studies of multiparametric monitoring (mostly a combination of standard remote monitoring and intrathoracic impedance) showed improved diagnostic yield in the detection of HF deterioration when this monitoring was applied. However, randomized studies showed controversial results regarding overall healthcare use when multiparametric monitoring was applied. One reason may be due to differences in monitoring between studies (e.g., different monitored parameters) as well as the definition of high-risk groups or positive alerts. Moreover, these studies were also different in the clinical approach after the detection of a positive alert. Although the initial observational studies suggested that patients with positive alerts had a higher risk of hospitalization, consequent randomized trials did not reproduce these results as key clinical outcomes such as mortality and HF hospitalization were not eventually lowered by multiparametric monitoring. One explanation could be the higher-than-expected variability of the monitored parameters in each patient, which makes the interpretation of these changes to a meaningful clinical conclusion challenging. Moreover, in a group of patients, the physiologic changes are temporal and resolve spontaneously without any intervention. These changes would go unnoticed if no monitoring was applied. Due to this complex interaction of physiologic parameters, it is almost impossible to expect that every alert-directed clinical action has a necessarily beneficial effect. This may be the reason that in general randomized trials could not show a significant reduction in clinical outcomes in the group with active multiparametric monitoring. The application of artificial intelligence in monitoring and interpretation of these highly changing physiologic data may therefore overcome this burden in the future. The initial results of two currently available algorithms, especially from the HeartLogic algorithm, seem promising as these algorithms incorporate longer intervals in the calculation of baseline values and each patient also represents their own control. Still, we have to wait and see the efficacy of these algorithms proven in randomized control trials to make a reliable conclusion. 

## 4. Conclusions and Future Directions

Timely detection of worsening HF provides an opportunity for appropriate intervention and therefore prevention of HF hospitalization and the possible prevention of cardiovascular and all-cause mortality. Because of the ever-increasing burden of HF hospitalization on individual patient quality of life and overall healthcare, CIED-based remote monitoring in HF patients seems a perfect opportunity to address worsening heart failure events well before potential hospitalization is needed. 

An abundance of physiological data are made available by CIEDs, without the need for the implantation of additional monitoring devices. However, this review has shown that even though an elegant theoretical association with pulmonary congestion and intrathoracic impedance measured from lead(s) to device, the diagnostic accuracy is still not good enough to be used as a sole marker for an impeding WHF event. 

Multiparameter algorithms showed great promise with improved diagnostic accuracy and time to clinical action as compared to intrathoracic impedance monitoring alone. However, inconsistent results from randomized intervention studies have taught great lessons on the application of CIED-enabled remote HF monitoring. Important lessons on study design, but also for use in clinical practice should be taken from the studies reviewed here: (1) patients selected should be at high risk for heart failure hospitalization, as diagnostic accuracy seems to be highest in these patients; (2) a standardized protocol for alert management, without including physician interpretation, or the patient itself in the alert pathway, should be in place to make sure that an impact on the endpoint can be timely instituted; finally, (3) the management protocol triggered by the alert should be instituted without needing the same justification as when a patient is presenting with HF in a traditional way, as this will deny any early intervention. 

Two device-based algorithms with integrated diagnostics seem very promising in meeting the diagnostic properties needed to be able to implement a successful clinical practice CIED-based remote HF monitoring program. These algorithms, however, still need further investigations on their possible role in the prevention of clinically worsening HF events. These investigations are currently underway and will hopefully soon provide the initial evidence that the evolution of CIED-based remote HF monitoring described in this review can be of added value to support clinical practice in the growing burden of HF care. 

## Figures and Tables

**Table 1:** Overview of available multisensor algorithms.

Boston Scientific	Medtronic
HeartLogic algorithm	Triage-HF algorithm
Heart sounds (first and third heart sounds and the ratio of the two)	Optivol^TM^ (intrathoracic impedance)
Thoracic impedance	Physical activity
Respiration rate	Night ventricular rate
Ratio of respiration rate to tidal volume	AF/Atrial tachycardia burden
Nightly heart rate	Ventricular rate during AF/AT
Physical activity	%CRT pacing
	Treated VT/VF
	Shocks

## Data Availability

No new data were created or analyzed in this study.

## Supplementary Materials

The following supporting information can be downloaded at: https://www.mdpi.com/article/10.3390/jcdd10040152/s1. Table S1: Observational studies using single and multisensory monitoring; Table S2: RCT’s using single- and multisensory monitoring.
