# Supplementary material for: Cardiovascular Implantable Electronic Devices Enabled Remote Heart Failure Monitoring; What We Have Learned and Where to Go Next
Source: J Cardiovasc Dev Dis. 2023 Mar 31;10(4):152. doi: 10.3390/jcdd10040152 (PMC10142884; doi:10.3390/jcdd10040152)
Supplement: Supplementary file 1 [file jcdd-10-00152-s001.zip › jcdd-2299897-supplementary.pdf]

Supplementary tables.

Table S1. Observational studies using single and multisensory monitoring

	No. patients	NYHA class	EF (mean)	Study design	Sensor monitoring	Adapted treatment after alarm	Primary endpoint	Effect of monitoring on the endpoints
MED-HeFT (2005)	34	II-IV	31%	Prospective	Uni-vector Intrathoracic impedance (Optivol)	No	HF hospitalization	Sensitivity: 76.9%, FPR: 1.5 per patient-year
FAST (2011)	155	II-IV	=<35%	Prospective	Univector intrathoracic impedance (Optivol)	No	HF hospitalization, HF emergency visit, unscheduled in-office visit	Sensitivity: 74%<br>FPR 2.08 per patient-year
InSync Snes (2007)	372	II-III	25%	Prospective	Univector Intrathoracic impedance (Optivol)	No	Worsening HF including pulmonary congestion, peripheral edema, worsening NYHA classification. Increased body weight	Sensitivity: 60%<br>PPV 60%
SENSE-HF (2011)	501 (371 completed	I-IV	26%	Prospective<br>(Phase I blinded)	Univector Intrathoracic	In phase II and III	<u>Phase I:</u><br>HF hospitalization with evidence of	<u>Phase I:</u><br>Sensitivity: 20.7% (dynamic sensitivity,

	phase II/III)				impedance (Optivol)		pulmonary congestion<br><br><u>Phase II/III:</u> worsening HF with evidence of pulmonary congestion	40.1% in patient with the longest interval between device implant and hospital admission)<br><br>PPV: 4.7%<br><br><u>Phase II/III</u><br><br>Sensitivity: 39.0%<br>PPV: 38.1%
DEFEAT-PE	144	II-III	25%	Prospective	Multi-vector intrathoracic impedance (Cor Vu)	No	Any hospitalization or emergency room visit for pulmonary congestion<br><br>or any urgent clinic visit requiring either intravenous diuretics<br><br>or vasoactive drugs for pulmonary congestion	Sensitivity: 21.6%<br><br>FPR: 0.9 per patient-year<br><br>PPV: 12.3

PARTNERS HF	694	III-IV	=<35%	Prospective	Multiparametric, univector intrathoracic impedence in combination with<br><br>AF duration, ventricular rate during AF, fluid index, patient activity, night heart rate, HRV, % of CRT pacing, ICD shocks for VT/VF.	No	HF hospitalization with pulmonary congestion	More HF hospitalization in patients with positive diagnostic, HR: 5.5, 95% CI: 3.4 to 8.8

Table S2. RCT ‘s using single- and multisensory monitoring

Study	Study design	Monitored data	Groups	Audible alerts	No. patients	NYHA Class	EF (mean)	Endpoints	Effect of monitoring on the end points

DOT-HF (2011)	Prospective, randomized	Intrathoracic impedance (could activate alarm), Cardiac Compass diagnostic (after patient-physician contact): trends on<br>heart rate variability, physical activity, arrhythmia incidence, percentage<br>ventricular pacing, and other diagnostic information. No remote monitoring.	Access arm vs. Control arm	On	335 patients (168 acces arm vs. 167 control arm)	II-IV	25%	Primary: combined all-cause mortality and HF hospitalization<br><br>Secondary:<br>a)All cause mortality<br>b)HF hospitalization<br>c) Number of outpatients visits<br>d) Percentage of OptiVol threshold crossings associated with signs and<br>symptoms of clinically relevant events.	Primary: 28% access arms vs 19% in control arm;<br>HR 1.52, CI 0.97–2.37, P 0.063<br><br>Secondary:<br>a) 115 in access arm vs 8% in control arm; HR 1.24; 95% CI, 0.63–2.44; P 0.54;<br>Figure 3B<br>b)35.7% in access arm vs 21.5% in control arm; HR 1.79; 95% CI, 1.08 –2.95; P 0.022<br>c) 250 in acces arm vs 84 in control arm, P<0.001<br>d) for HF hospitalization HR 3.02 (1.52-5.98), P<0.001

									for outpatient visits HR 6.78 (4.03-11.4), P<0.001
OptiLink (2016)	Prospective, multicenter, randomized, unblinded	Remote monitoring via Medtronic CareLink (including Optivol). 75% of alerts were transmitted.	Intervention group (transfer of fluid index alerts followed by protocol specific intervention) vs control group	No audible alerts for the patients	1002 patients (505 intervention group vs. 497 control group)	NYHA II-III	26.7%	Primary: composite of all-cause death and CV hospitalization<br><br>Secondary:<br>-all-cause mortality<br>-cardiovascular<br>mortality<br>- composite of all-cause death and HF hospitalization<br>-CV hospitalizations -HF hospitalizations -all-cause hospitalizations	Primary:<br>18 and 24 months, 59.0%, 52.7% in the intervention group vs 56.1%, 47.8%) in control group, HR, 0.87, CI 0.72–1.04,P= 0.13<br><br>After adjustment for prior HF hospitalization or IV diuretic: HR 0.84, CI 0.70–1.02, P=0.07<br>Secondary:<br>All p values >0.05<br><br>After adjustment for prior HF hospitalization or IV diuretic:<br>- combined all caurse mortality and HF

									hospitalization P=0.03, other secondary outcomes P>0.05
TRUST (2010)	Prospective, multicenter, randomized	Automatic remote monitoring of arrhythmia and device-specific parameters	HM vs. conventional	No	1339 (908 HM va. 431 conventional)	NYHA II-IV	29%	1st primary: No total in-hospital device evaluation<br>2nd primary: adverse event rates (death stroke, events requiring surgical intervention<br>Secondary: detection time of clinically significant problem	1st primary: 2.1 vs 3.8 per patient year in HM vs conventional (P<0.001)<br>2nd primary: Survival 96.4% (CI 95.5%-97.6%) in HM vs 94.2% (CI 91.8%- 96.6%) in the conventional group (P=0.174)<br>Stroke 0.3 vs 1.2 in HM vs conventional (P=0.120)<br>Surgical evaluation 6.6 vs. 4.9% in HM vs. conventional (P= 0.269)<br>Secondary:

									Median 1 d vs 35.5% in HM vs conventional (P<0.001).
CONNECT (2011)	Prospective, multicenter, randomized.	Remote monitoring of arrhythmia and device-related alerts, via Medtronic CareLink. 55% succesful transmissions	Remote monitoring vs. standard in-clinic visits	On, Audible alerts for the patient off.	1997 patients (1014 remote arm va. 983 in-office arm)	NYHA II-IV	28.9%	Primary: time from clinical event to clinical decision<br><br>Secondary: cardiovascular health care utilization	Primary: lower in remote arm vs in-office arm<br><br>Secondary: no difference, however lower total rate of in-clinic visits due to replacement of in between visits with remote monitoring in remote arm.
EVOLVO (2012)	Prospective, multicenter, randomized, open	Remote monitoring via Medtronic CareLink (evaluation of intrathoracic impedance, atrial arrhythmia, delivered ICD shocks)	Remote monitoring via CareLinks vs. standard care using audible alerts	On	200 patients (remote arm vs. standard arm)	NYHA II-IV	31%	Primary: all emergency department visits and urgent<br>in-office visits<br><br>Secondary:<br>- visits related to<br>episodes of worsening of HF	Primary: 75 in remote arm vs 117 in standard arm, IRR 0.65, CI 0.49–0.88; P<0.005

								- visits for<br>arrhythmias or ICD-related episodes<br>- rate of total healthcare use	
IN-TIME (2014)	Prospective, multicenter, randomized	Telemonitoring data sent to Biotronik Home Monitoring Service Center (Ventricular and atrial tachyarrhythmia, low percentage of biventricular pacing, increase in the frequency of ventricular extrasystoles, decreased patient activity, abnormal intracardiac electrogram)	Telemonitoring vs. standard care	N/A	664 patients (333 telemonitoring group vs. 331 control group	NYHA II-III	26%	Primary:<br>Worsening of a composite clinical score (death, overnight admission for worsening of heart failure, worsened of NYHA functional class and worsened self-assessment<br>Secondary: -all cause mortality<br>-hospital admission because of worsening heart failure	Primary: 18.9% in telemonitoring group vs. 27.2% in control group (OR 0·63, 95% CI 0·43–0·90, P=0.013)<br>Secondary:<br>-all cause mortality; 3.4% in telemonitoring group vs. 8.7% in control group (HR 0·36, 95% CI 0·17–0·74, P=0.004)<br>- hospital admission for worsening of heart failure: 44 in telemonitoring group vs. 47 in control group, P=0.38)

Luthje et al (2015)	Prospective, single-Center, randomized	Remote monitoring via Medtronic CareLink (including Optivol)	Remote monitoring via CareLink vs. standard care	On, no audible alerts	176 patients (87 remote group vs. 89 control group)	NYHA I-IV	31.9%	Primary: time to first HF-related hospitalization	Primary: HR 1.231 ([0.621–2.438]; P= 0.551
MORE-CARE (2017)	Prospective, multicenter, randomized	Remote monitoring via Medtronic CareLinks (including Optivol), 88.2% successful transmission	Remote arm via CareLink vs. standard arm	On for Optivol, atrial arrhythmia and device integrity. Audible alert for the patient off	865 patients (437 remote arm vs. 428 to standard arm	NYHA II-IV	27.3%	Phase 1: time from device-detected event to clinical decision<br><br>Phase 2:<br>-primary: combined all-cause mortality and CV and device-related hospitalization.<br>-Secondary: a)the utilization of healthcare resources for CV reasons<br>b) the number of hospitalizations, ED admissions, and outpatient visits separately;	Phase 1<br><br>Phase 2:<br><br>-primary: 28.7% in remote arm vs 34.3% in standard arm (P=0.89)<br><br>-secondary<br>a) IRR 0.62 (0.58–0.66), P <0.001,<br>b)<br>-all cause hospitalization: IRR

								c) the<br><br>costs related to utilization of healthcare resources for CV and device<br><br>reasons<br><br>d) the safety of RM in CRT-D patient management.	1.02 (0.83-1.26), , P=0.83<br><br>-ED admissions: IRR 0.72 (0.53–0.98), P=0.037<br><br>-outpatient visits: IRR 0.59 (0.56–0.62), P<0.001<br><br>c)Cost saving of remote arm €2899 per 100 patients at 2 years<br><br>d) AER 55 in remote arm vs 53 in standard arm, P=0.92
REM-HF (2017)	Prospective, multicenter, randomized, open-label	Remote monitoring different manufacturers	Remote monitoring vs usual care	Off	1650 patients (824 remote monitoring, 826 usual care)	NYHA II-IV	29.9%	Primary: 1st<br><br>event of the composite of death fromany cause or an unplanned hospitalization<br><br>for cardiovascular reasons<br><br>Secondary:<br><br>-death	Primary: 42.4% in remote group vs 40.8% usual care group, HR 1.01, CI 0.87-1.18, P=0.87<br><br>Secondary: all p-values >0.05

								from any cause<br>-cardiovascular death<br>- non-cardiovascular death<br>-cardiovascular-related death or unplanned cardiovascular hospitalization<br>- death from any cause or unplanned hospitalization for noncardiovascular<br>Reason<br>-unplanned cardiovascular hospitalization<br>- unplanned hospitalization for non-cardiovascular reasons.	
